# Polymeric nanomaterials encapsulating fluorescent polyindole-nido- carborane: design, synthesis and biological evaluation

**DOI:** 10.3389/fchem.2024.1402640

**Published:** 2024-07-05

**Authors:** Jia Cao, Tao Jin, Shihe Shao, Boneng Mao, Jin Feng

**Affiliations:** ^1^ Endoscopy Center, Department of Gastroenterology, Shanghai East Hospital, School of Medicine, Tongji University, Shanghai, China; ^2^ Medical College of Anhui University of Science and Technology, Huainan, China; ^3^ Department of Gastroenterology, Yixing People’s Hospital Affiliated to Jiangsu University, Yixing, China

**Keywords:** BNCT, polymeric nanomaterial, nido-carborane, encapsulating, cell image

## Abstract

The water-soluble nido-carborane was prepared by alkali treatment of o-carborane. A polymer PInd containing a polyindole structure was synthesized and employed to label the modified *o*-carborane. Subsequently, four polymeric nanomaterials were synthesized with the objective of encapsulating them in order to enhance its bioavailability. The experimental results showed that the fluorescent complex encapsulated by the pH-sensitive polymer A had the best UV absorption and fluorescence intensity, and thus A-PInd-C was chosen for subsequent experiments. The Transmission electron microscopy images revealed that the compounds exhibited a rounded internal morphology, with the layers exhibiting a tightly stacked arrangement. The AFM imaging revealed that the surface of the sample exhibited a relatively uniform and smooth appearance. *In vitro* release experiments conducted under acidic conditions demonstrated that A-PInd-C was released in a predominantly linear manner, with a maximum release rate of 80% observed within 48 h. Cellular imaging experiments showed that the compound could enter HeLa and HCT-116 cells and was mainly distributed around the nucleus, especially in the acidic environment. The results of the cell proliferation toxicity experiments demonstrated that A-PInd-C exhibited inhibitory effects on HeLa, PC-3 and L02 cells. Among these, the inhibitory effect on PC-3 cells was the most pronounced, reaching up to 70%. In conclusion, this paper solves the problem of poor bioavailability of carborane by improving the boron containing compounds and also makes the system have potential for Boron neutron capture therapy.

## Graphical Abstract

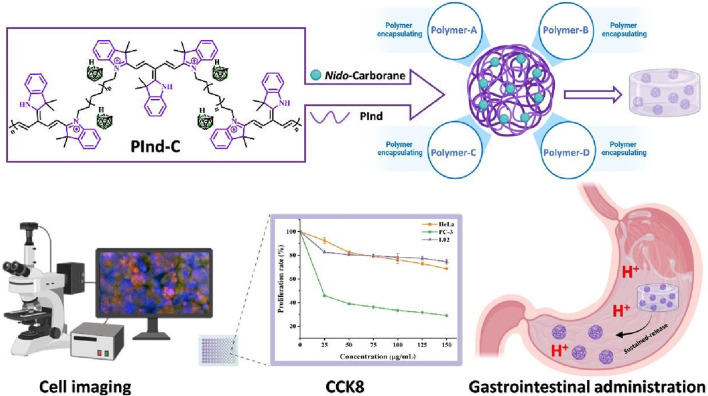

## 1 Introduction

Boron neutron capture therapy (BNCT) is a highly targeted and selective treatment that has been demonstrated to be effective in the cure of various types of cancers while causing less damage to healthy cells. (Suzuki, 2020; He et al., 2021; Sauerwein et al., 2021). Currently, the focus of BNCT research is concentrated on the search for novel boron drugs that are highly effective and have good tumor specificity (Barth et al., 2018; Dymova et al., 2020; Hu et al., 2020; Coghi et al., 2023). Carboranes (Wang et al., 2021; Chen et al., 2022; Marfavi et al., 2022) are the products of substitution of boron atoms by carbon atoms in polyhedral borane molecules. These can be classified into two main groups: closed and open. Among the products of the disintegration of the roof of the closed carborane under the action of strong alkali is *nido*-carborane (Fox et al., 2002), which not only has the similar geometrical configuration and high boron content with carborane, but also has excellent water solubility. Therefore, *nido*-carborane has a great potential for application in the field of BNCT therapy (Lee et al., 2020; Shao et al., 2021; Varaksin et al., 2021; Gruzdev et al., 2022; Gruzdev et al., 2023).

Nanomedicine is the application of the principles and methods of nanoscience and technology to medicine, which has more advantages in terms of stability, targeting and pharmacokinetics than traditional pharmaceutical dosage forms. (Li and Kataoka, 2021; Wen et al., 2023). Polymeric nanomaterials have a wide range of applications in the field of drug encapsulation (Thakral et al., 2013; Villemin et al., 2019; Joy and John, 2022; Lages and Nicolas, 2023). They have excellent solubility properties and very high stability, and at the same time are non-toxic and non-irritating, which can effectively improve the drug delivery efficiency. By encapsulating drugs with polymers, it can realize the release, diffusion and play a role in the active site in a specific physiological environment (Cardoso et al., 2023; Liu et al., 2023), thus solving the problems of ineffective dissolution and difficult absorption of fat-soluble drugs during drug delivery due to the shortcomings of poor aqueous solubility, low selectivity, low bioavailability and so on of many drugs (Jain et al., 2020; Patriota et al., 2021; Real et al., 2022).

Indole is a nitrogen heterocyclic compound containing a π-conjugated system, and its derivatives are widely distributed in nature and employed in various fields, including material science, biomedicine, and so forth. (Yuan and Jia, 2018; Sun et al., 2023). Indole fluorescent dyes have certain structural modification possibilities and excellent fluorescent properties due to the aromatic benzene ring structure and the five-membered ring structure with heteroatoms in their skeleton (Kathiravan et al., 2021; Singh et al., 2022). Their large molar extinction coefficient, good stability, solubility and non-toxicity have led to their extensive use in various scientific fields, including biological labelling and the development of new fluorescent dyes. (Sun et al., 2020; Beser et al., 2022; Gomes et al., 2023; Liu et al., 2023; Lian et al., 2023).

In this paper, we combine the above aspects to address the poor bioavailability of carborane. We modify the fat-soluble *o*-carborane into a water-soluble *nido*-carborane by alkali treatment. Additionally, we synthesize a polyindole fluorescent dye to label the *nido*-carborane. Finally, four polymeric nanomaterials were designed and synthesized for encapsulation with the objective of enhancing the biocompatibility of the drug and achieving a slow release in different gastrointestinal environments. This research aims to enable the development of simple and practical boron-containing fluorescent drugs as well as to improve the functionality of nanomedicine.

## 2 Experiment

### 2.1 Materials and instruments

All the reagents and solvents used in this experiment were purchased from regular sources and can be used directly without purification. The infrared spectra were determined by the potassium bromide press method on a Nicoletavato-370FT-IR analyzer. UV and fluorescence spectra were recorded by UV-2550 spectrophotometer and Shimadzu RF-5301PCS, respectively. Transmission electron microscopy (TEM) was done by HT7800 120 KV transmission electron microscope. Zeta potential and particle size were measured by Zetasizer Nano ZS90. All the tests were carried out at room temperature.

### 2.2 Synthesis

#### 2.2.1 Synthesis of Polymers-A, B, C and D

Methyl methacrylate (MMA), Butyl methacrylate (BMA), Diethylaminomethyl methacrylate (DEAMEA) were added to the round-bottom flask in appropriate proportions, and then THF was added and stirred to 60°C. Then, the THF solution of AINB was added in one time, and the solution was removed after stirring at 60°C for 1h, and white granular solid Polymers-A (MMA: BMA: DEAMEA = 1:1:2) was obtained. Polymers-B (MMA: EA = 1:1) is prepared by the same method.

MMA, Ethyl Acrylate (EA), (Methacrylatoethyl trimethyl ammonium chloride) MTC were added to the round-bottom flask in appropriate proportions, and then anhydrous ethanol was added and stirred to 60°C. Then add Dimethyl 2,2′-azobis (2-methylpropionate) (AIBME) anhydrous ethanol solution at one time, stirring at 70°C for 1h, at which time it becomes a colorless viscous liquid. The reaction was continued in the water bath at 70°C for 4h, and finally the transparent and yellowish hard solid Polymers- C (MMA: EA: MTC = 1:1:0.2) and Polymers-D (MMA: EA: MTC = 1:1:0.1) were obtained. (Liu et al., 2023).

#### 2.2.2 Synthesis of 2-(3,3-dimethylindolin-2-ylidene) malonaldehyde

Add 2.75 mL DMF to a round-bottled flask, stir in an ice water bath for 5 min, then add 1.44 mL of POCl3 and continue stirring for 20 min. A DMF solution (1g/2.5 mL) of 2,3, 3-Trimethyl-3H-Indole is then dropped into the mixed system. Remove the reaction bottle from the ice bath and continue stirring at 75°C for 5 h. Ice water is then added and the pH is adjusted to neutral with NaOH(aq). After standing for 24 h, the precipitation was filtered and recrystallized in ethanol after air drying to obtain yellow crystals. (Li et al., 2023).

#### 2.2.3 Synthesis of compound 3

2,3, 3-trimethyl-3h-Indole (5.04 mL, 31.4 mmol) and 3-Bromoprop-1-ene (4.08 mL, 47.1 mmol) were dissolved in ACN (20 mL), then the reaction was stopped after stirring at 80°C for 20 h. The reaction was confirmed by thin layer chromatography. Rotational evaporation ACN, silica gel column chromatography (silica gel:200–300 mesh, eluent: PE:AE = 1:5) was used for isolation and purification. The blue oily product three is obtained.

#### 2.2.4 Synthesis of PInd

Compound 2 (0.1 g) and compound 3 (0.5 g) were dissolved in EtOH(4 mL) with 25 μL of piperidine. Then the reaction was stopped after heating and stirring at 85°C for 3 h. The reaction was confirmed by thin layer chromatography. The solvent is evaporated by rotation and the purple product PInd is obtained.

#### 2.2.5 Synthesis of nido-carborane and PInd-C

The o-carborane (0.5 g, 3.57 mmol) and KOH (0.2 g, 3.56 mmol) were dissolved in 3 mL of EtOH and stirred at 80°C for 1 h. The EtOH was rotary evaporated to give the white solid *nido*-carborane. (Wang et al., 2024).

PInd (0.1 g) and *nido*-carborane (0.1 g) were dissolved in 3 mL of MeOH and the ion-exchange reaction (Ouyang et al., 2023) was stirred at 50°C for 2h, and KCl was removed by hot filtration to obtain the product PInd-C.

#### 2.2.6 Preparation of encapsulated compound PInd-C by polymers (preparation of A-PInd-C, B-PInd-C, C-PInd- C and D-PInd-C)

The PInd-C (50 mg) was dissolved in 2 mL of THF, Polymers-A (50 mg) was added, and heat stirred for 3 h. The THF was rotary evaporated, resulting in solidification of the nanoparticles. The A-PInd-C was collected by centrifugation (8,000 rpm, 20 min) and washed 3 times with deionized water. The B-PInd-C, C-PInd-C and D-PInd-C were all obtained using the same method.

### 2.3 The collection of UV–vis spectra and fluorescence spectra

The sample to be tested was dissolved in ethanol to prepare a mother solution with a suitable concentration. Then, the UV and fluorescence spectra of PInd-C were measured in six different solvents (DCM, DMSO, (Ethyl Acrylate)AE, THF, MeOH, and EtOH). In addition, the UV and fluorescence spectra of A-PInd-C, B-PInd-C, C-PInd-C, and D-PInd-C were measured in phosphate buffer solutions with pH values of 4.5, 5.5, and 6.5 (DMSO was added to enhance the solubility of the four polymers, with a ratio of PB:DMSO = 50:1, V:V). Finally, the fluorescence stability of these polymers was also determined.

### 2.4 *In vitro* release test

First, prepare phosphate buffers with pH values of 4.5, 5.5, and 6.5, and add 5% sodium dodecyl sulfate (SDS, w/v) to each buffer. Next, measure the absorbance of PInd-C in each buffer at a wavelength of 582 nm, and plot a standard curve of sample concentration absorbance. Then, dissolve the sample to be tested in the three surfactant-containing phosphate buffers mentioned above to prepare a 2.0 mg/mL drug-loaded micelle solution. Take 2.0 mL of the drug-loaded micelle solution and add it to a dialysis bag (MD34-1000000), which is then placed in a 20 mL release medium. The release experiment is conducted under constant temperature oscillation (100 rpm/min) at 37 °C. Samples are taken at 1, 2, 3, 4, 6, 8, 10, 14, 18, 22, 28, 36, and 48 h, with a sampling volume of 3 mL, while 3 mL of fresh buffer is added. Measure the absorbance of the release medium at 582 nm at each time point, and calculate the content of PInd-C in the release medium based on the standard curve. Finally, calculate the cumulative release of.

PInd-C according to the following formula.

Q%=CnV+Vi∑i=0n−1Ci dmrug×100%

Q: Percentage of cumulative drug release, %

C_n_: Concentration of the *n*th sample taken, μg/mL.

V: Total volume of release medium, 20 mL.

V_i_: Sampling volume at time point i, 3 mL.

C_i_: Concentration of the sample taken at time point i, μg/mL

m_drug_: Quality of C-IR775 in drug-loaded micelles, μg.

### 2.5 Zate potential and particle size testing

Dissolve the sample to be tested in ethanol, and use ultrasound for 5 min. Then add deionized water to a total volume of 5 mL. Continue ultrasonic treatment for 15 min to ensure uniform dispersion of the sample. Subsequently, each sample is tested on a nanoparticle size potentiometer to obtain Zate potential and particle size data. Each sample is measured three times to obtain accurate results.

### 2.6 Cell imaging

Hela and HCT-116 cells in logarithmic growth phase were seeded into 12-well cell culture plates, with 5 × 10^^^4 cells per well and cultured for 24 h. Subsequently, A-PInd-C was diluted with DMSO to a stock solution of 100 mg/mL, and the sample was diluted with culture medium to a final concentration of 50 μg/mL. After the diluted sample was prepared, the original cell culture medium was removed, washed with PBS, and 2 mL of the diluted sample solution was added to the cell culture plate. The pH was adjusted to 50 μg/mL with 0.1 mol/L hydrochloric acid. After continuing to culture for 24 h, an appropriate amount of DIPA (final concentration of 5 μg/mL) was added, and the cell culture solution was removed. 500 μL of diluted dye solution was added to each well. Finally, we incubated in a 37°C incubator for 10–15 min, observed the staining effect under a fluorescence microscope, and selected a suitable wavelength for photography under a confocal microscope.

### 2.7 Cell proliferation toxicity test (CCK8)

LO2, HeLa and PC-3 cells in the logarithmic growth phase were selected and digested by pancreatic enzymes to prepare cell suspension. According to the experimental requirements, the concentration of cell suspension was adjusted, and 5 × 10^^^4 cells per well were inoculated into the 96-well plate, with three compound pores in each group. Based on the experimental grouping conditions, each group of cells was subjected to sample addition treatment. Among them, the blank control group was not treated with sample addition. In the experimental group, A-PInd-C solution of 25 μg/mL, 50 μg/mL, 75 μg/mL, 100 μg/mL and 125 μg/mL were added, and the cells were co-cultured for 24 h. Then, 96-well plates were taken out, 10 μL CCK8 solution was added to each hole, and incubated in the incubator for 1 h. Finally, the absorbance at 450 nm was measured by enzyme-linked immunoassay.

## 3 Results and discussion

### 3.1 Design

**SCHEME 1:**
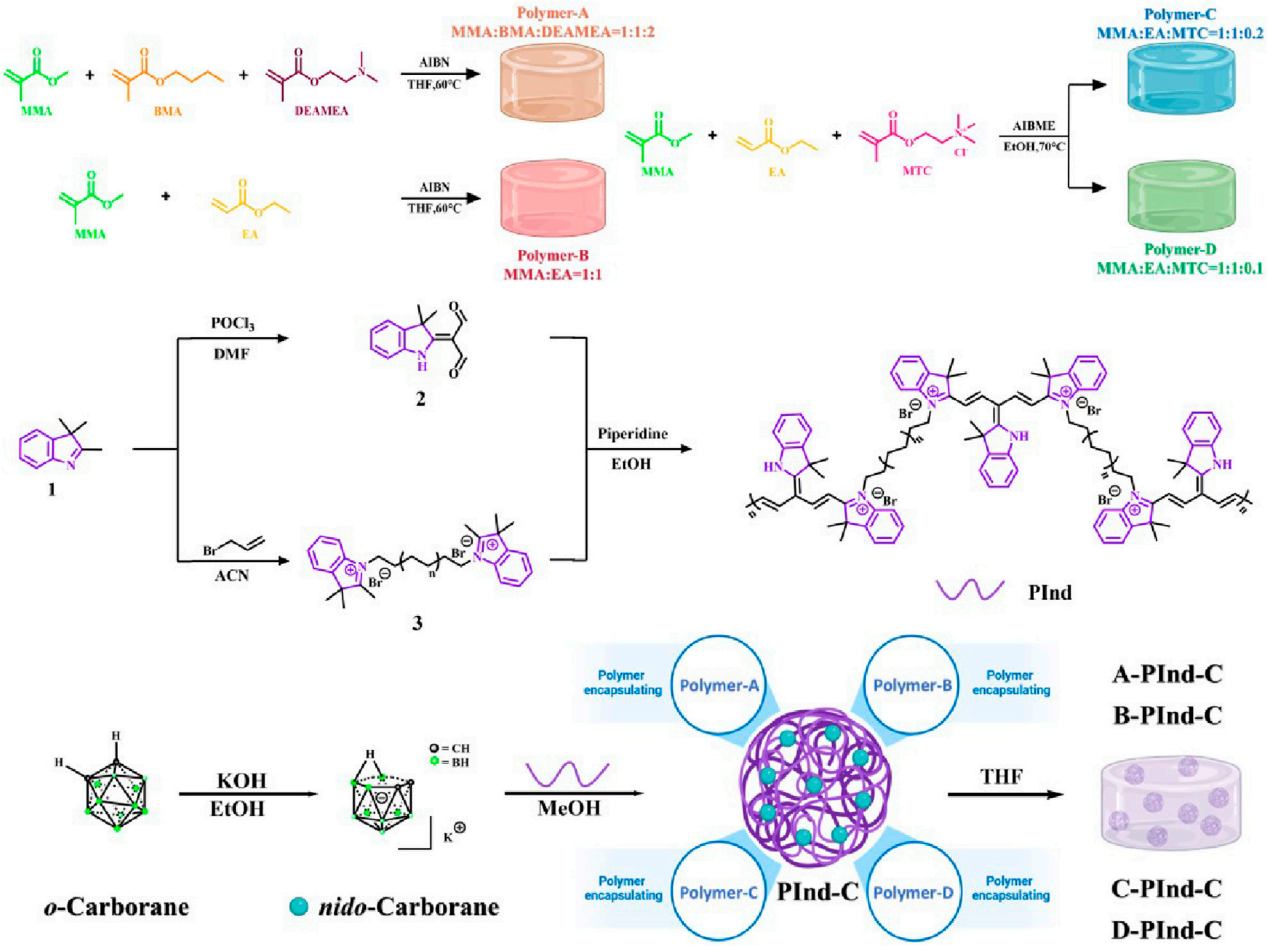
Preparation method of **Pind-C** encapsulating by Polymers (Polymer-A, Polymer-B, Polymer- C and Polymer-D, respectively).

The polymer encapsulation technique is mainly used for the delivery of poorly soluble drugs. In this paper, four different types of polymers were first designed and synthesized for subsequent encapsulation of compounds. Then, using 2,3, 3-Trimethyl-3H-Indole as the initial material, while the compounds were made to have two aldehyde groups by the Vilsmeier-Haack reaction. On the other side, the electrophilic reagent 3-Bromoprop-1-ene is used to attack the electron-rich N atom in the indole ring to form the quaternary ammonium salt. The two were then linked by a condensation reaction to produce a polyindole quaternary ammonium salt PInd with a pirlindole structure. At the same time, the *o*-carborane is treated with a base to obtain the potassium salt of the *nido*-carborane, which has a good water solubility. And binds to PInd by ion exchange. Finally, four previously synthesized polymers were encapsulated to obtain four fluorescent polymers with good biocompatibility and selectivity. (Scheme 1).

### 3.2 Characterization of A-PInd-C, B-PInd-C, C-PInd-C and D-PInd-C

**FIGURE 1:**
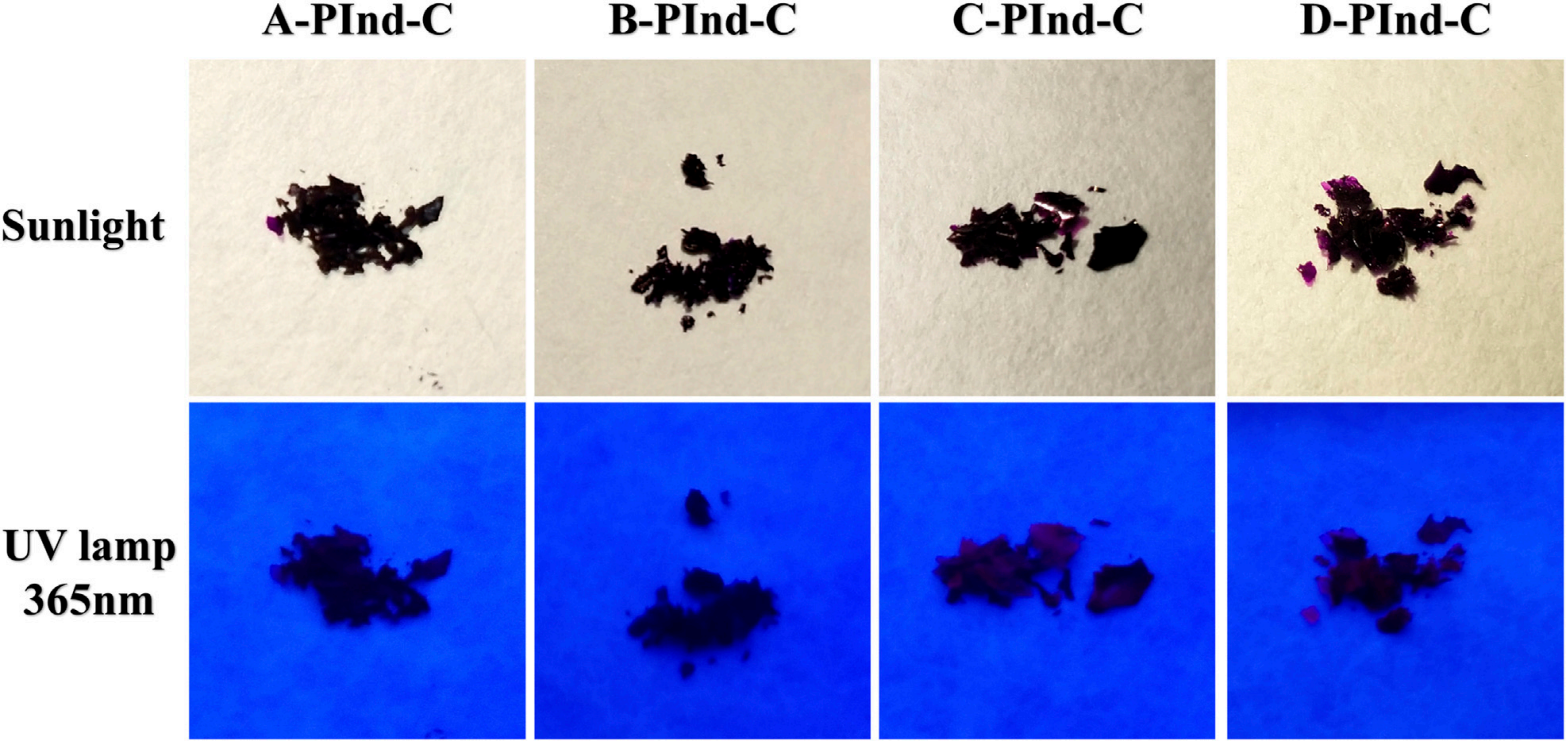
Photographs of ***A-PInd-C, B-PInd-C, C-PInd-C and D-PInd-C*** exposed to sunlight and a 365- nanometer ultraviolet lamp.

As shown in Figure 1, four fluorescent polymers were prepared in this experiment. Under natural light, all four substances showed dark purple clumps. Due to the dark and purple color of the samples, most of the samples appeared black and only the edge part showed purple when observed under UV light.

**FIGURE 2:**
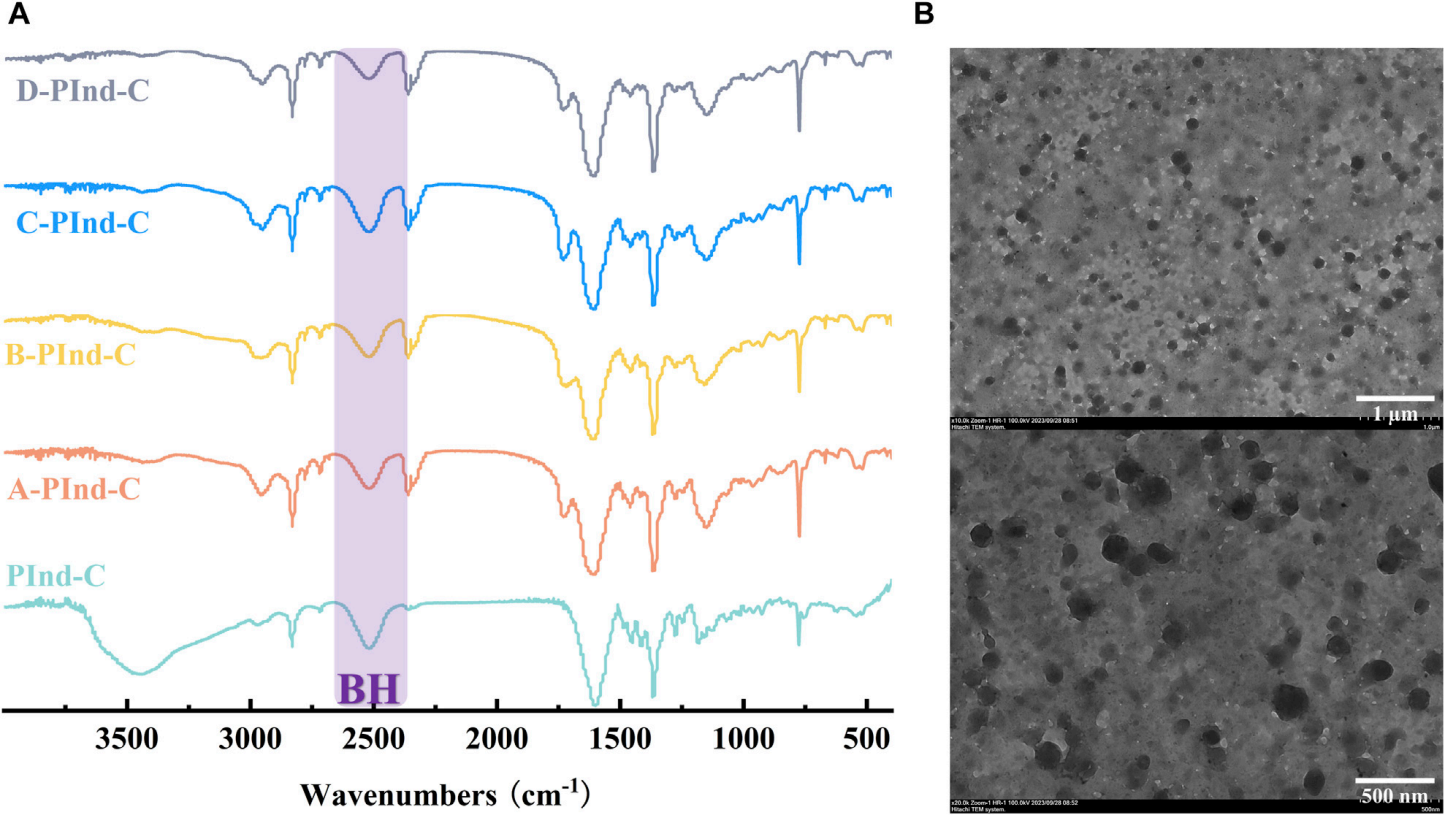
**(A)** FT-IR spectra of **Pind-C**, ***A-PInd-C, B-PInd-C, C-PInd-C and D-PInd-C***. **(B)** Representative TEM images of ***A-PInd-C***.

The structural properties of PInd-C and four fluorescent polymers were analyzed by infrared spectroscopy (as shown in Figure 2A). It can be clearly observed from the figure that the five substances all show a significant infrared absorption peak at 2,500 cm^-1^, which corresponds to the stretching vibration of the B-H bond, which is a unique characteristic peak of nido-carborane. From this, we can be sure that the nido-carborane molecule has successfully bound to PInd. In addition, we can also observe that PInd- C has a unique infrared absorption peak that is different from the other four polymers near 3,500 cm^-1^, which indicates that the four polymers have successfully encapsulated PInd-C.

In order to further explore the micromorphological characteristics of the sample and the encapsulation status of the drug, we selected A-PInd-C with good properties for TEM testing according to the following experimental considerations. As shown in Figure 2B, sample PInd-C is circular in shape, closely stacked together, and completely enveloped by Polmer-A. By means of ImageJ, we measured the diameter of compound PInd-C from TEM images and plotted its particle size distribution. The average particle size of PInd-C was 98.1 nm. At the same time, we also measured the average particle size of A-PInd-C using DLS technology and found that the average particle size was 650.1 nm. This is also consistent with the dense stacking of PInd-C layers seen in the TEM.

### 3.3 Spectroscopic properties

**FIGURE 3:**
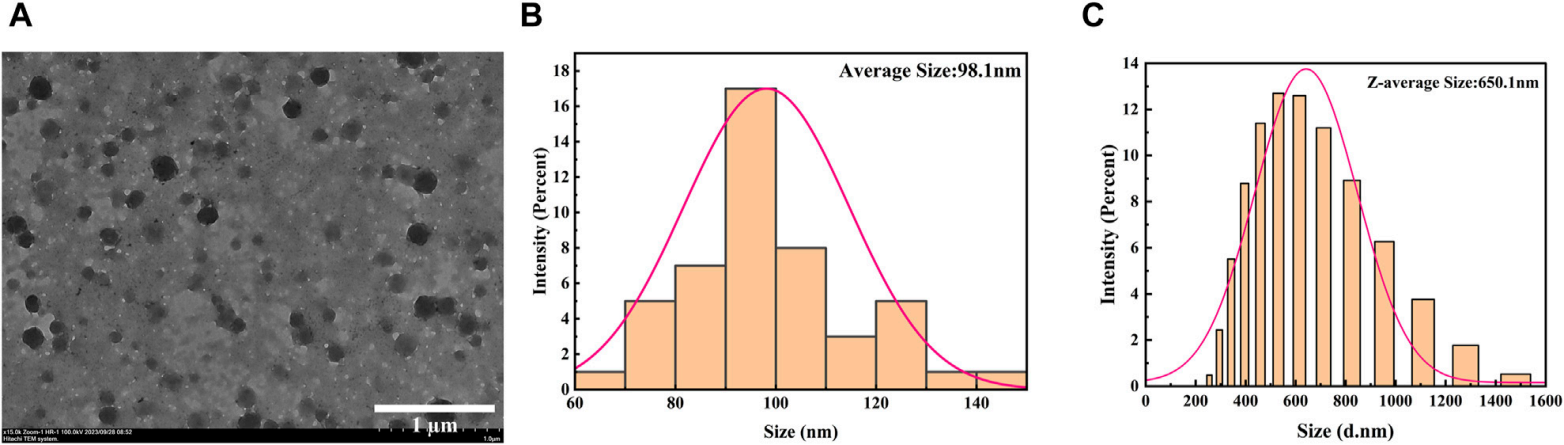
**(A)** TEM images of ***A-PInd-C***. **(B)** The size distribution of **PInd-C** measured by ImageJ in TEM image. **(C)** Size distribution by DLS of **A-PInd-C**.

**FIGURE 4:**
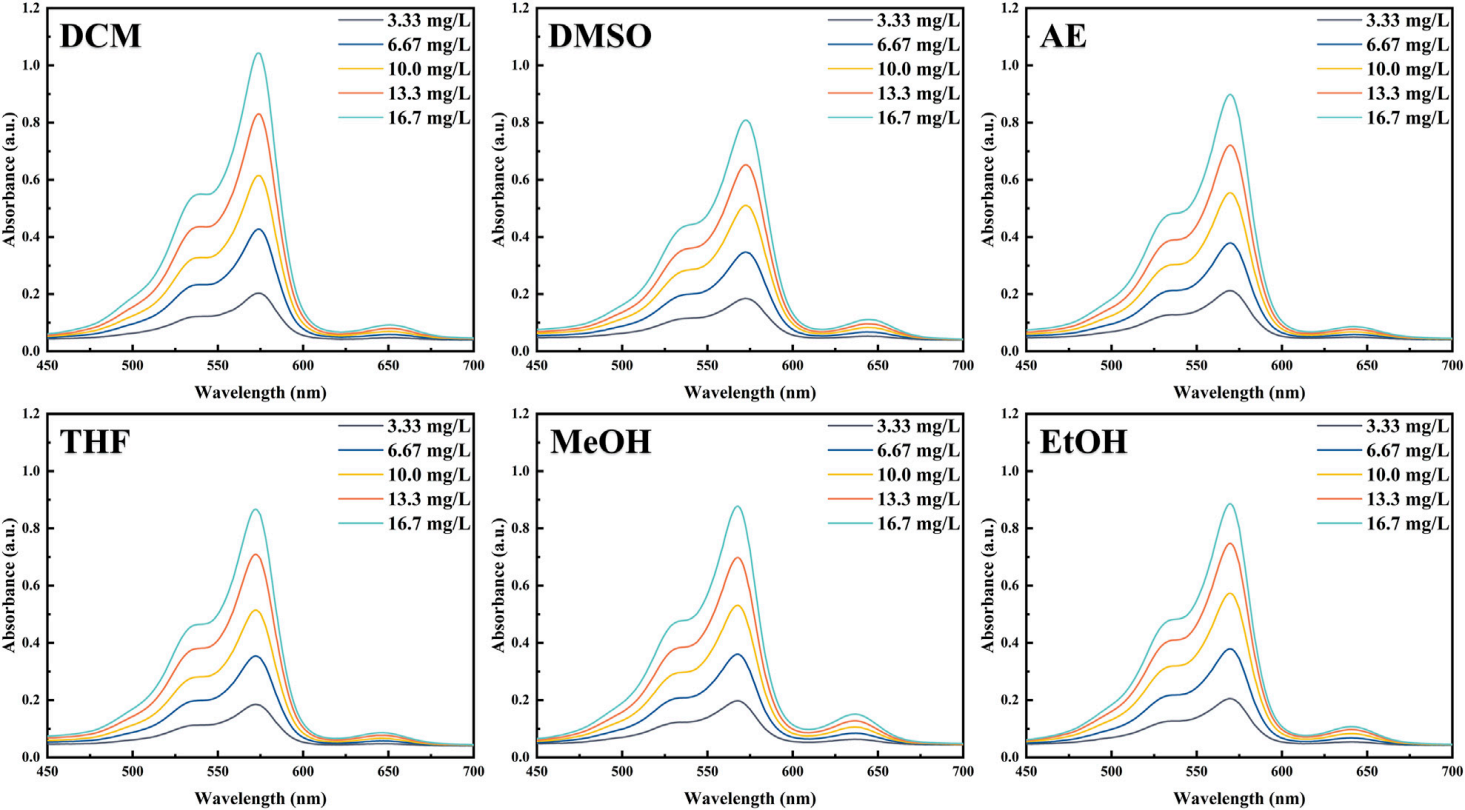
The absorption spectra of **Pind-C** in different solvents.

**FIGURE 5:**
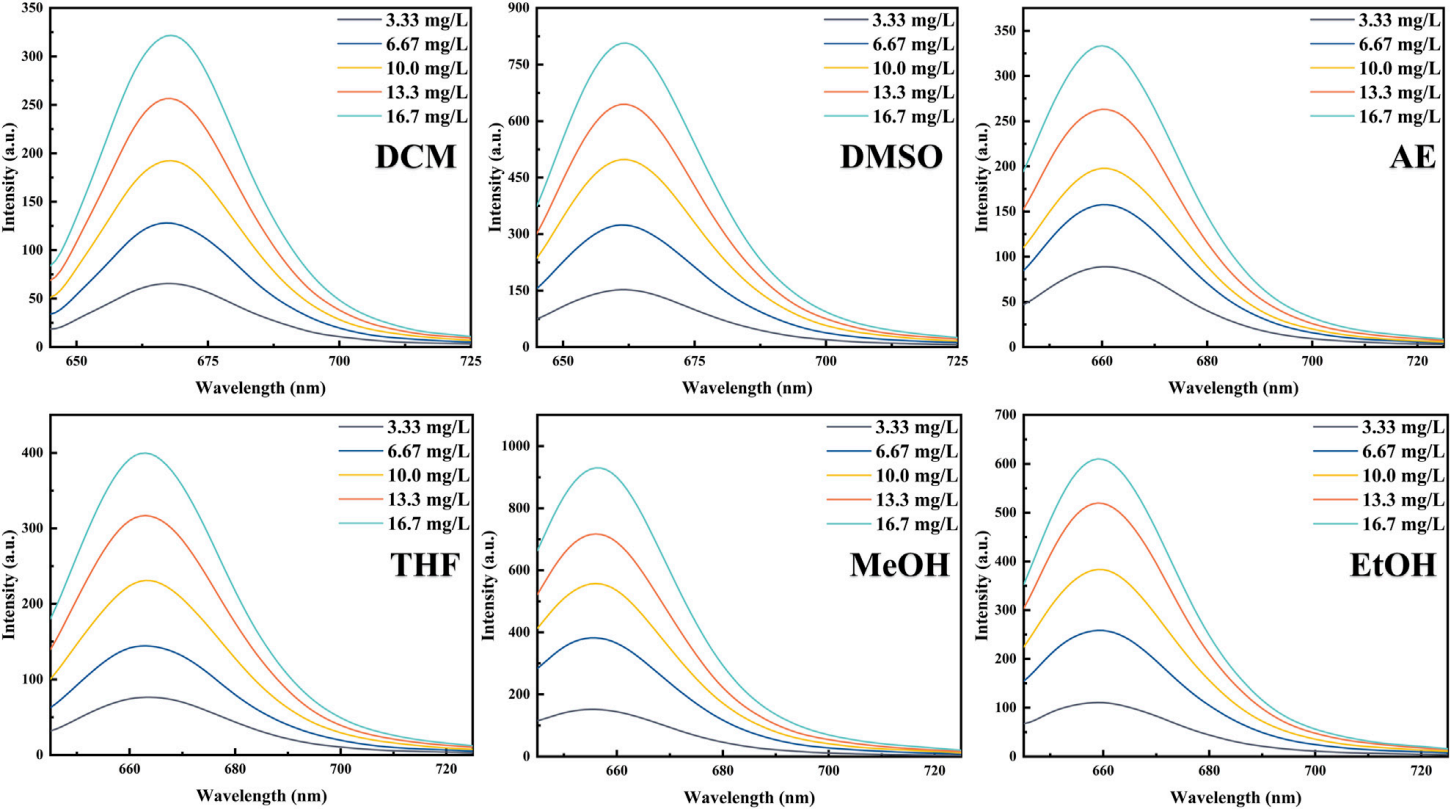
The emission spectra of **Pind-C** in different solvents.

To investigate the photophysical properties of PInd-C, we selected six solvents with different polarities, including DCM, DMSO, AE, THF, MeOH, and EtOH, and performed UV and fluorescence spectroscopy measurements on it. The experimental results indicate that among these six solvents, the UV spectrum of PInd-C exhibits identical double-peak characteristics within the range of 525–580 nm, with its maximum absorption peak located between 567 and 574 nm. Under the same concentration conditions, PInd- C exhibits the highest absorbance in the DCM solvent, while the absorbance in the other five solvents remains relatively consistent. Additionally, the emission wavelengths of PInd-C in the six solvents are concentrated within the range of 656–668 nm. In particular, PInd-C shows the highest fluorescence absorption intensity in the MeOH solvent. Furthermore, we observed that both the absorbance and fluorescence intensity of PInd-C increase with the increase of concentration. (Figures 3–5; Table 1).

**TABLE 1:** Photophysical characteristic of **C-IR775** in different solvents.

Compound	Items	Solvents					
		DCM	DMSO	AE	THF	MeOH	EtOH
**Pind-C**	λ_abs_/nm	574	573	570	571	567	569
	λ_em_/nm	668	662	660	663	656	659
	Δ_max_ν, cm^-1^	2,451	2,346	2,392	2,430	2,393	2,400

**FIGURE 6:**
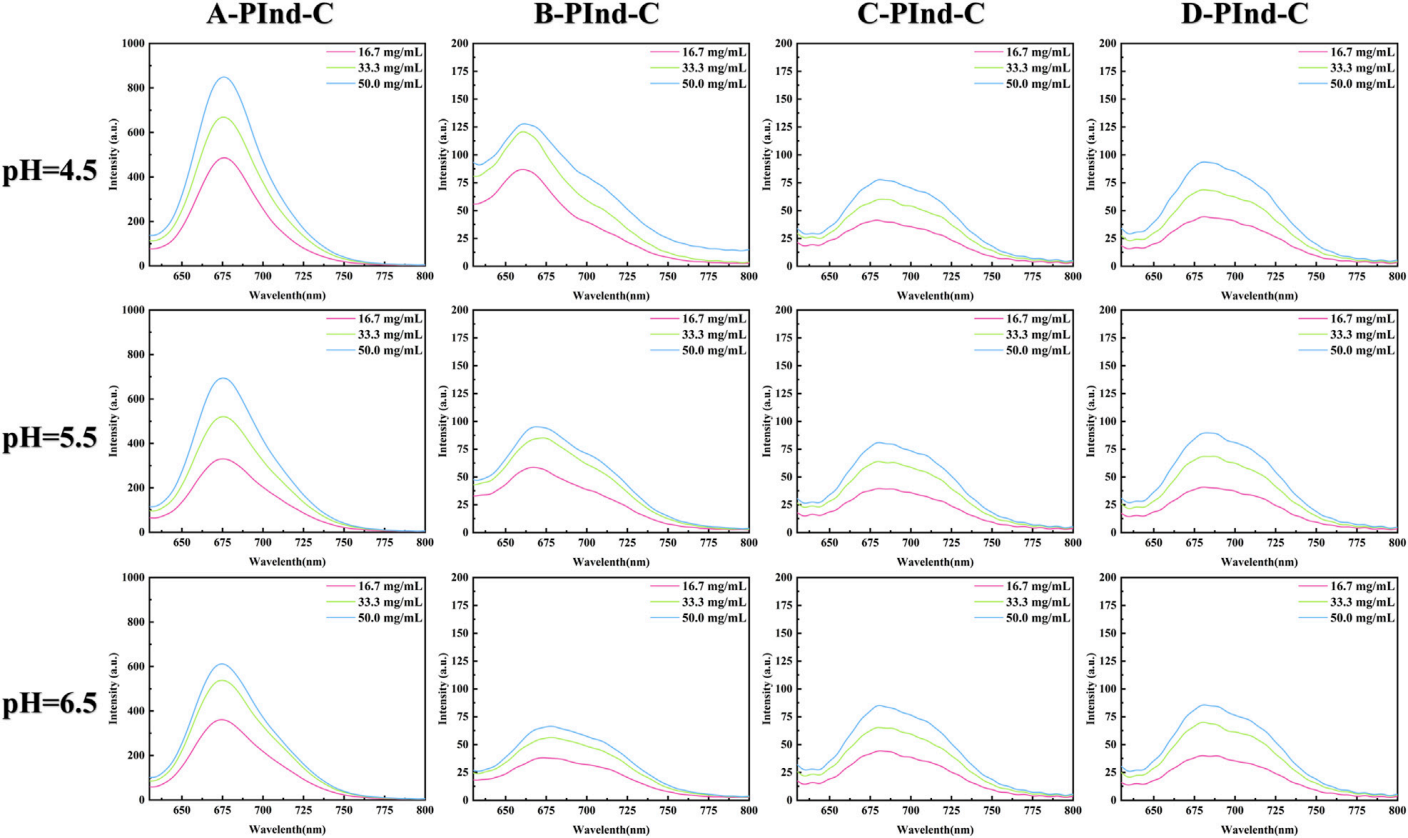
The absorption spectra of ***A-PInd-C, B-PInd-C, C-PInd-C and D-PInd-C*** in different pH solutions.

**FIGURE 7:**
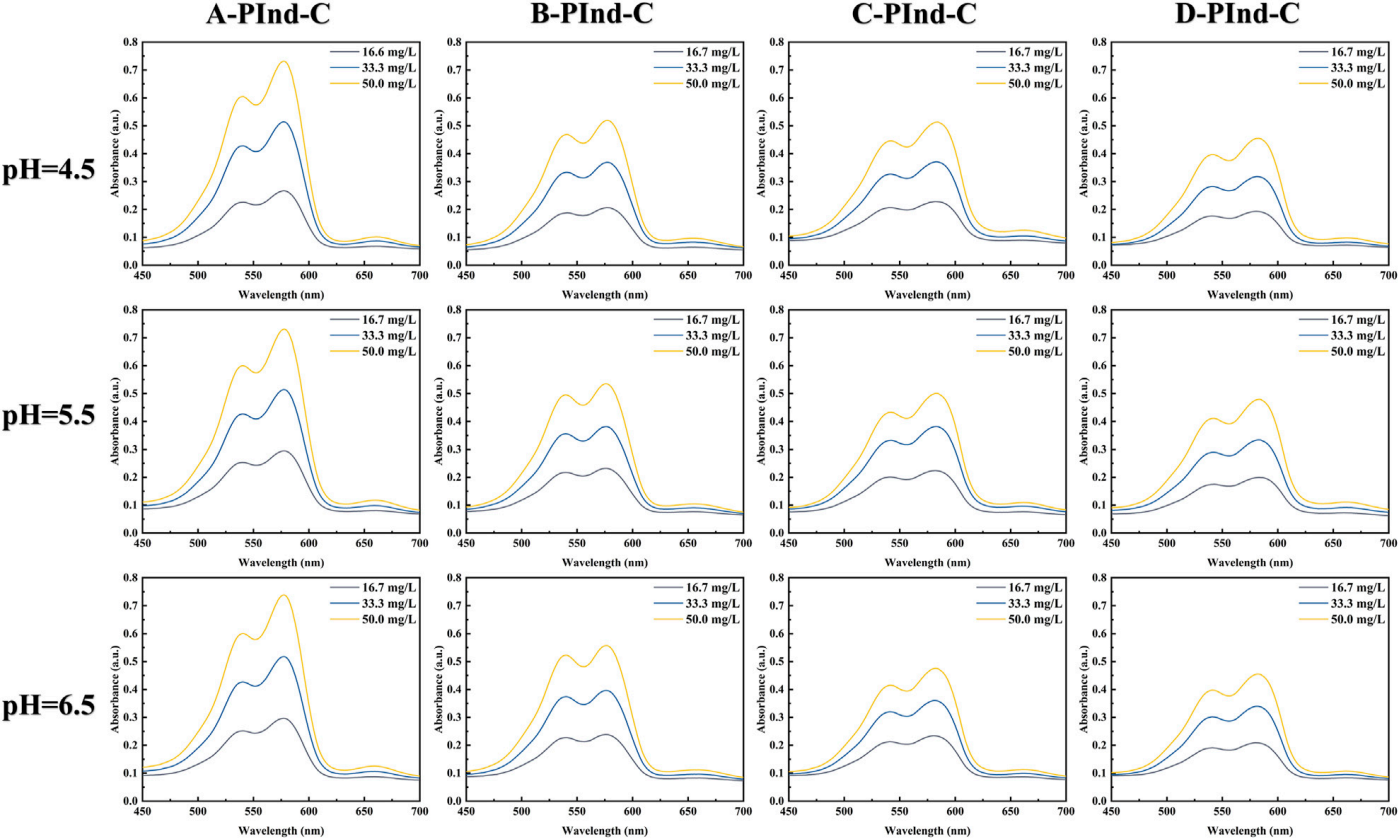
The emission spectra of ***A-PInd-C, B-PInd-C, C-PInd-C and D-PInd-C*** in different pH solutions.

**FIGURE 8:**
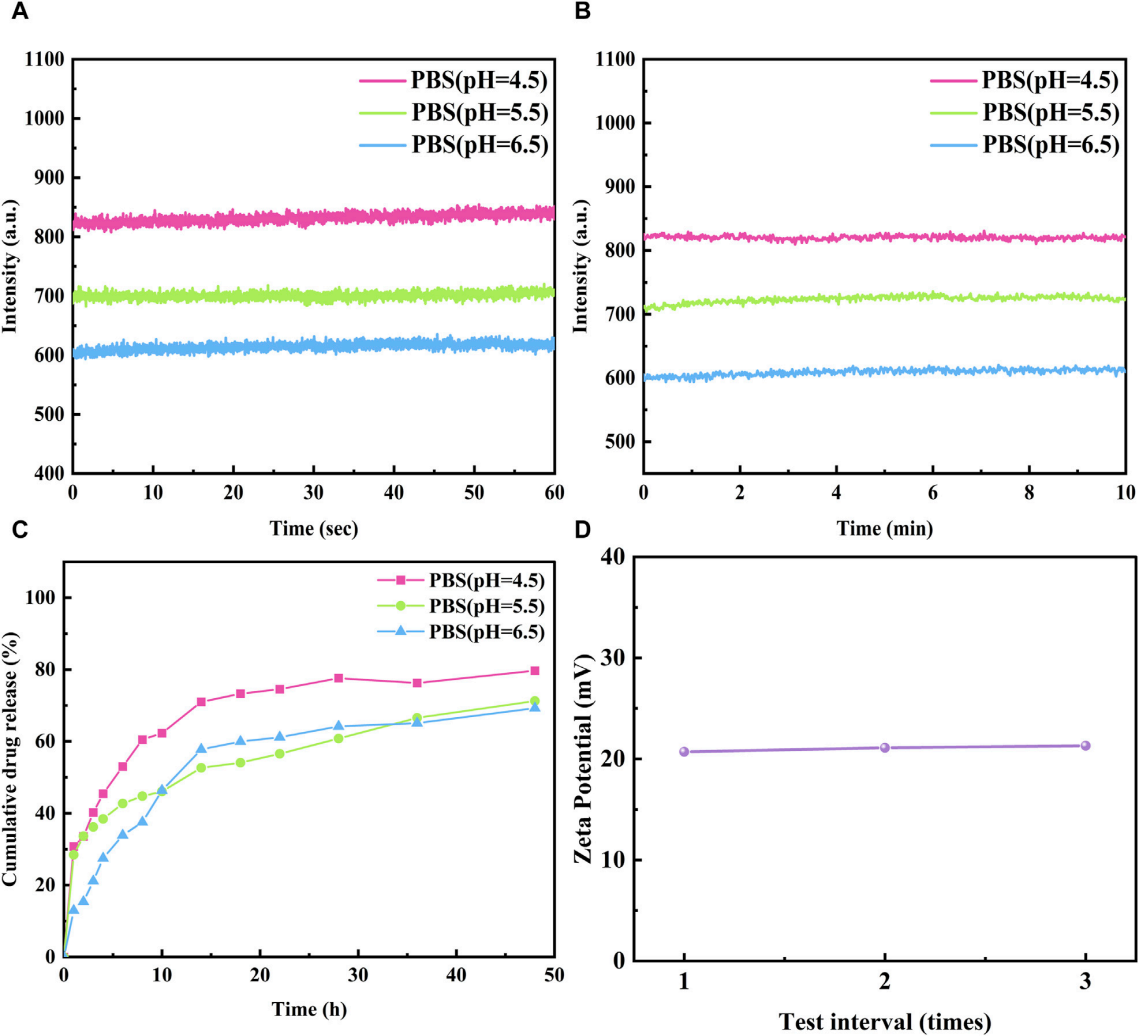
The fluorescence stability of **A-Pind-C** in different Phosphate Buffer **(A)** 60 s and **(B)** 600 s (Excitation was at 580 nm, emission at 675 nm). **(C)** Drug release of **A-Pind-C** at different pH. **(D)** The Zeta potential values of **A-Pind-C** in aqueous solution.

In order to investigate the photophysical properties of the four fluorescent polymers under different environments in the gastrointestinal tract, Phosphate Buffer with pH 4.5, 5.5 and 6.5 was selected for UV and fluorescence detection. According to Figures 6, 7, Table 2, the UV spectra of the four fluorescent polymers in different environments were relatively similar, all of them showed the same double-peak characteristics in the range of 535–585 nm, and the maximum absorption peaks were located in the range of 576–584 nm. Compared with PInd-C, the absorbance of the right main peak of the four fluorescent polymers is slightly decreased, which is due to the polymer wrapping. In addition, the emission wavelengths of the four fluorescent polymers were concentrated between 661–682 nm. From their fluorescence spectra, it is obvious that the fluorescence of A-PInd-C is stronger, and the fluorescence intensity gradually decreases with the increasing pH. This indicates that Polmer-A is a pH-sensitive polymer. In addition, the absorbance and fluorescence intensity of these polymers increased with increasing concentration. We also tested the fluorescence stability of these four fluorescent polymers as shown in Figures 8A, B. The fluorescence intensity of A-PInd-C was almost unaffected by time in three different pH environments; while the fluorescence intensity of the other three samples varied to some extent with time. Comprehensively comparing the UV and fluorescence performances of the four fluorescent polymers, the UV absorption effect and fluorescence effect of A-PInd-C were significantly better than those of the other three fluorescent complexes. Therefore, the compound A-PInd-C was mainly selected for the subsequent experiments.

**TABLE 2:** Photophysical characteristic of ***A-PInd-C, B-PInd-C, C-PInd-C and D-PInd-C*** in different Phosphate Buffer.

Compound	Items	Phosphate buffer		
		pH = 4.5	pH = 5.5	pH = 6.5
**A-Pind-C**	λ_abs_/nm	577	578	578
	λ_em_/nm	676	675	674
	Δ_max_ν, cm^-1^	2,538	2,486	2,464
**B-Pind-C**	λ_abs_/nm	578	576	576
	λ_em_/nm	661	665	670
	Δ_max_ν, cm^-1^	2,174	2,324	2,436
**C-Pind-C**	λ_abs_/nm	583	583	582
	λ_em_/nm	679	680	680
	Δ_max_ν, cm^-1^	2,425	2,447	2,476
**D-Pind-C**	λ_abs_/nm	582	584	582
	λ_em_/nm	680	682	680
	Δ_max_ν, cm^-1^	2,476	2,460	2,476

### 3.4 *In vitro* release

In order to evaluate the release behavior of A-PInd-C in the gastrointestinal environment, Phosphate Buffer at pH 4.5, 5.5 and 6.5 was selected for *in vitro* release experiments. As shown in Figure 8C, the release profiles of A-PInd-C in different pH environments all showed a classical three-stage pattern. In the first phase of the first 8 h, the drug experienced a rapid release phase; the next 8–24 h was the second phase, in which the drug release slowed down but still showed a certain release rate; and the last 24–48 h was the third phase, in which the drug release was further reduced and entered a slow-release phase. After 48 h, the drug release basically stops. It is noteworthy that the total amount of A-PInd-C release was maximum when the pH was 4.5, suggesting that it has the best effect in acidic environment. This result is consistent with the UV fluorescence spectral profile of A-PInd-C, further confirming its excellent performance in acidic environment.

### 3.5 Zeta potential

The stability of the A-PInd-C dispersion system *in vivo* was evaluated using the potentiometric assay technique. As shown in Figure 8D, the surface of A-PInd-C was positively charged with a potential of 21.0 ± 0.2 mV. This result indicates that the dispersion system has good stability, which helps the fluorescent polymer to enter into the tumor cells in the form of nano-dispersions, and thus is more conducive to the accumulation of the drug in the tumor site.

### 3.6 AFM

**FIGURE 9:**
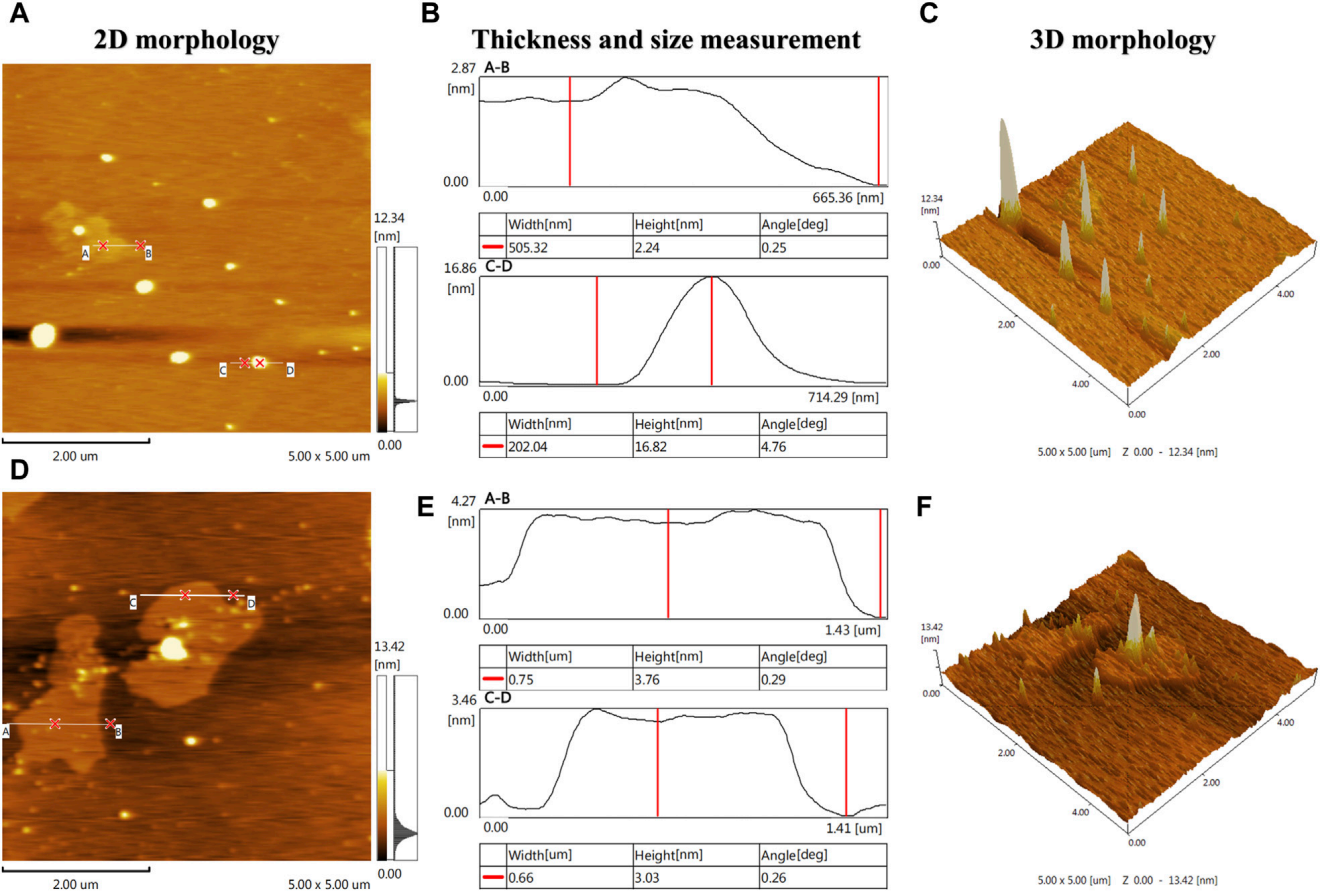
AFM imaging was performed at two locations on the **A-Pind-C**. [**(A, D)** shows the 2D morphology of the sample, **(B, E)** shows the thickness and dimensional measurements of the sample, and **(C, F)** shows the 3D morphology of the sample, where **(A, B)** is a set and **(D, E, F)** is a set].

In order to assess the stability and large-area homogeneity of A-PInd-C, AFM imaging was performed at two locations of the sample. According to the 2D morphology Figures 9A, D of the two different locations of the sample, most of the locations on the surface of the sample present a relatively uniform and smooth shape. According to the 3D morphology Figures 9C, F, there are some gullies and protrusions in the local area of the sample surface. The height of these protrusions is mainly concentrated between 2 and 17 nm (Figures 9B, E).

### 3.7 Cell imaging

**FIGURE 10:**
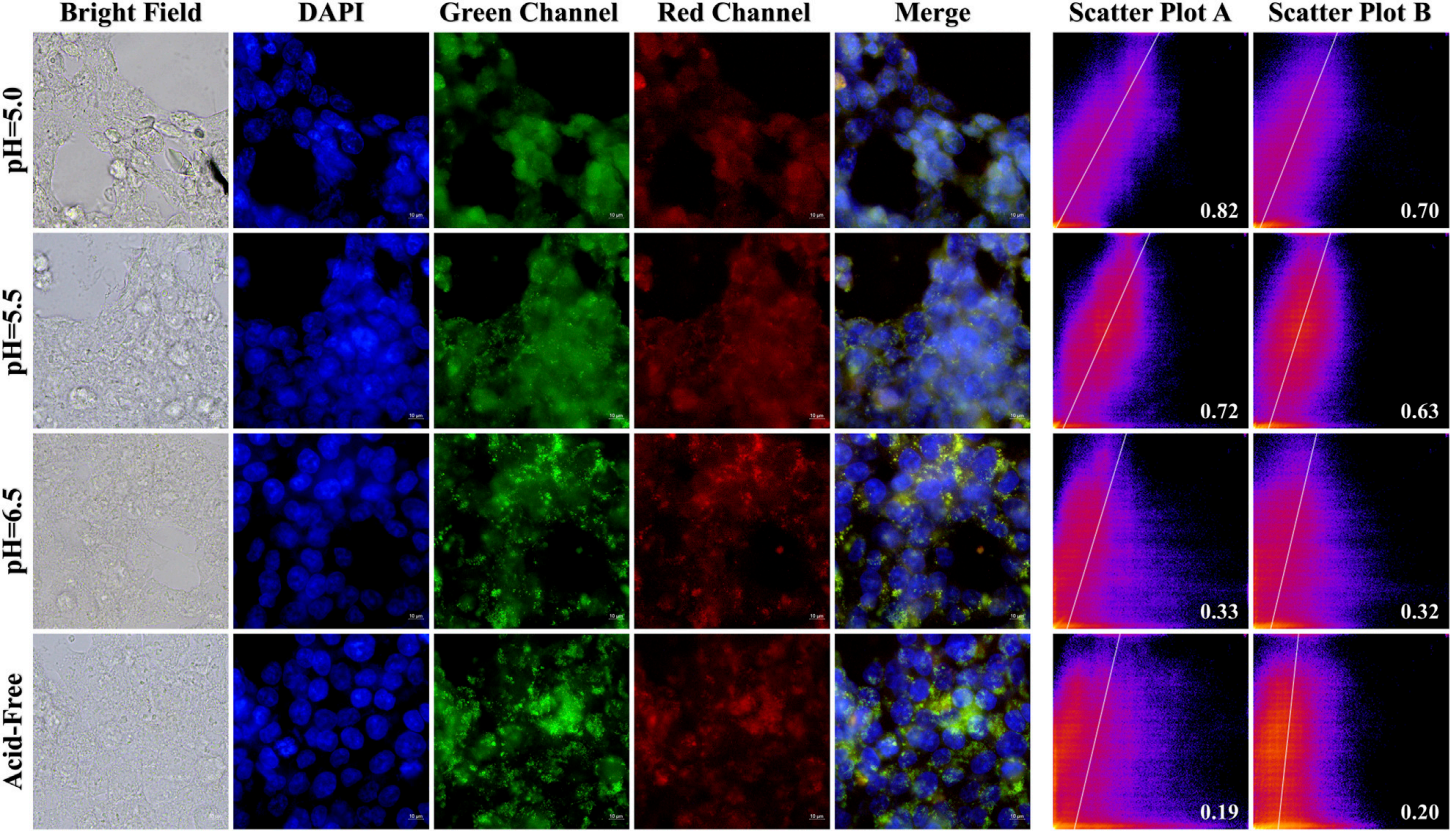
HeLa cells were imaged by fluorescence microscopy as well as 2D intensity histograms plotted with red, green and DAPI, respectively. (Sample concentration: 50 μg/mL) Scatterplot A shows the intensity scatterplot of the green channel with DAPI, and scatterplot B shows the scatterplot of the red channel with DAPI. (Insert: Pearsons’ correlation coefficient).

**FIGURE 11:**
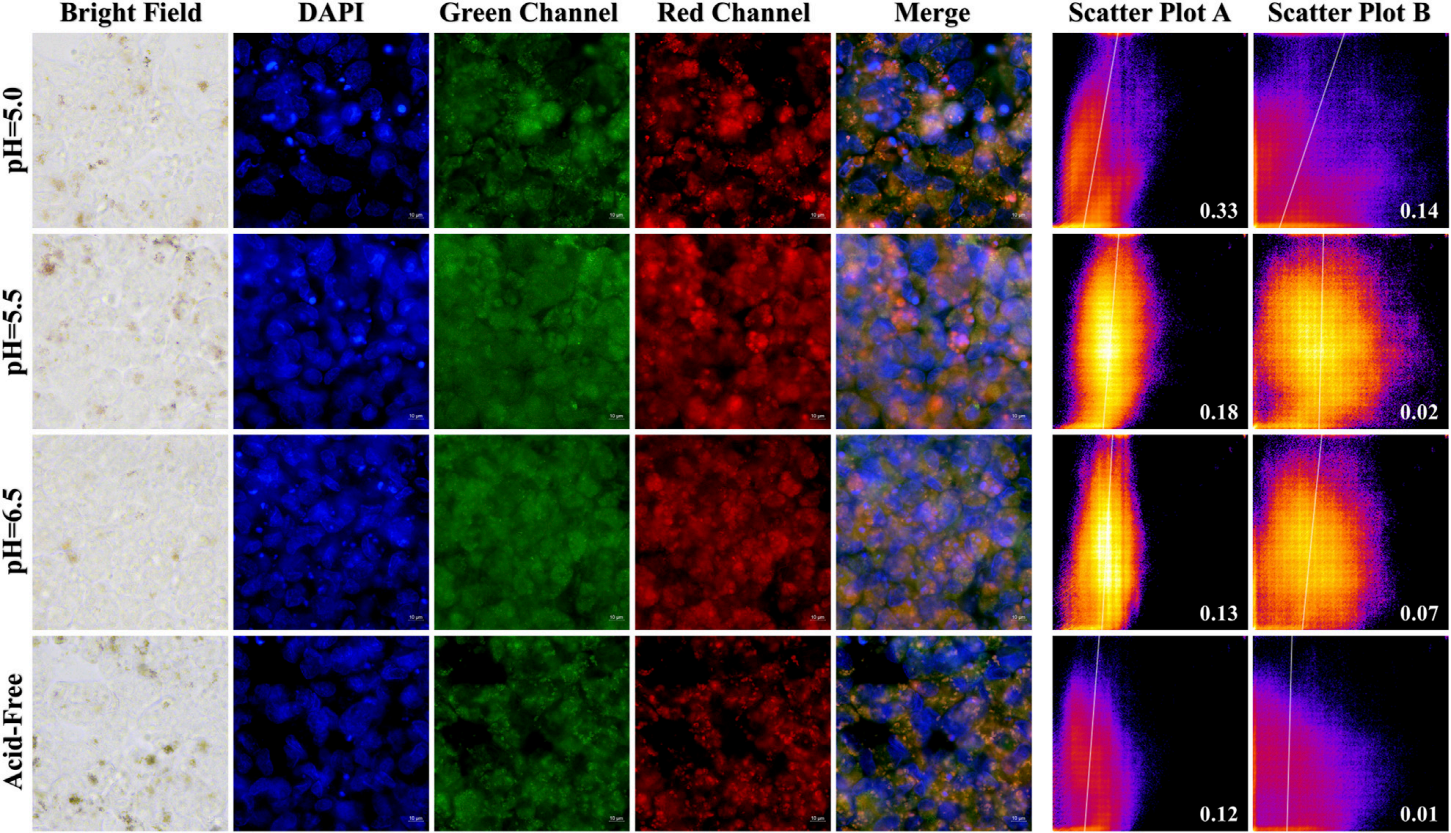
HCT-116 cells were imaged by fluorescence microscopy as well as 2D intensity histograms plotted with red, green and DAPI, respectively. (Sample concentration: 50 μg/mL) Scatterplot A shows the intensity scatterplot of the green channel with DAPI, and scatterplot B shows the scatterplot of the red channel with DAPI. (Insert: Pearsons’ correlation coefficient).

In order to investigate the selectivity and biocompatibility of the drug, we performed cell fluorescence imaging experiments on two cancer cells, Hela and HCT-116, in acidic and acid-free environments, respectively. During the experiments, we compared three acidic and acid-free environments, and also provided imaging of bright field, DAPI, green channel, red channel, and the latter three channels combined. As shown Figures 10, 11, A-PInd-C can pass through the cell membrane of both cancer cells and distribute around the nucleus. Especially in Hela at pH = 5.0, the compound fluorescence almost completely overlapped with the nucleus, indicating that most of the drug had entered the nucleus. In order to more accurately analyze the binding ability of A-PInd-C to cancer cells, we performed fluorescence co-localization analysis of the blue-green channel and blue-red channel using the software ImageJ. By plotting 2D intensity histograms, and calculating the Pearson’s coefficient, from the results we found that the binding ability of A-PInd-C to HeLa was much better than that of HCT-116 cells. Meanwhile, the binding ability of A-PInd-C to both cancer cells increased with decreasing pH, especially in Hela at pH = 5.0, the fluorescence of the compound almost completely overlapped with the nucleus of the cell, indicating that most of the drug had already entered the nucleus of the cell. In addition, all calculated Pearson’s coefficients were greater than 0, with a maximum of 0.82. This indicates that DAPI correlates to a certain extent with both the red and green channels, most of the compounds enter into the cell and are distributed in and around the cell, and a portion of the compounds can enter into the nucleus. Since Polymer-A is a stomach-soluble coated polymer, A-PInd-C has excellent biocompatibility under acidic conditions in both cells, which is also reflected in the imaging results. In summary, combining boron-containing compounds with fluorescent substances and then using polymer-coated materials can greatly improve the bioavailability of the compounds and achieve the desired results. More importantly, the system can improve the function of nanomedicine.

#### 3.8 Cell proliferation toxicity test (CCK8)

**FIGURE 12:**
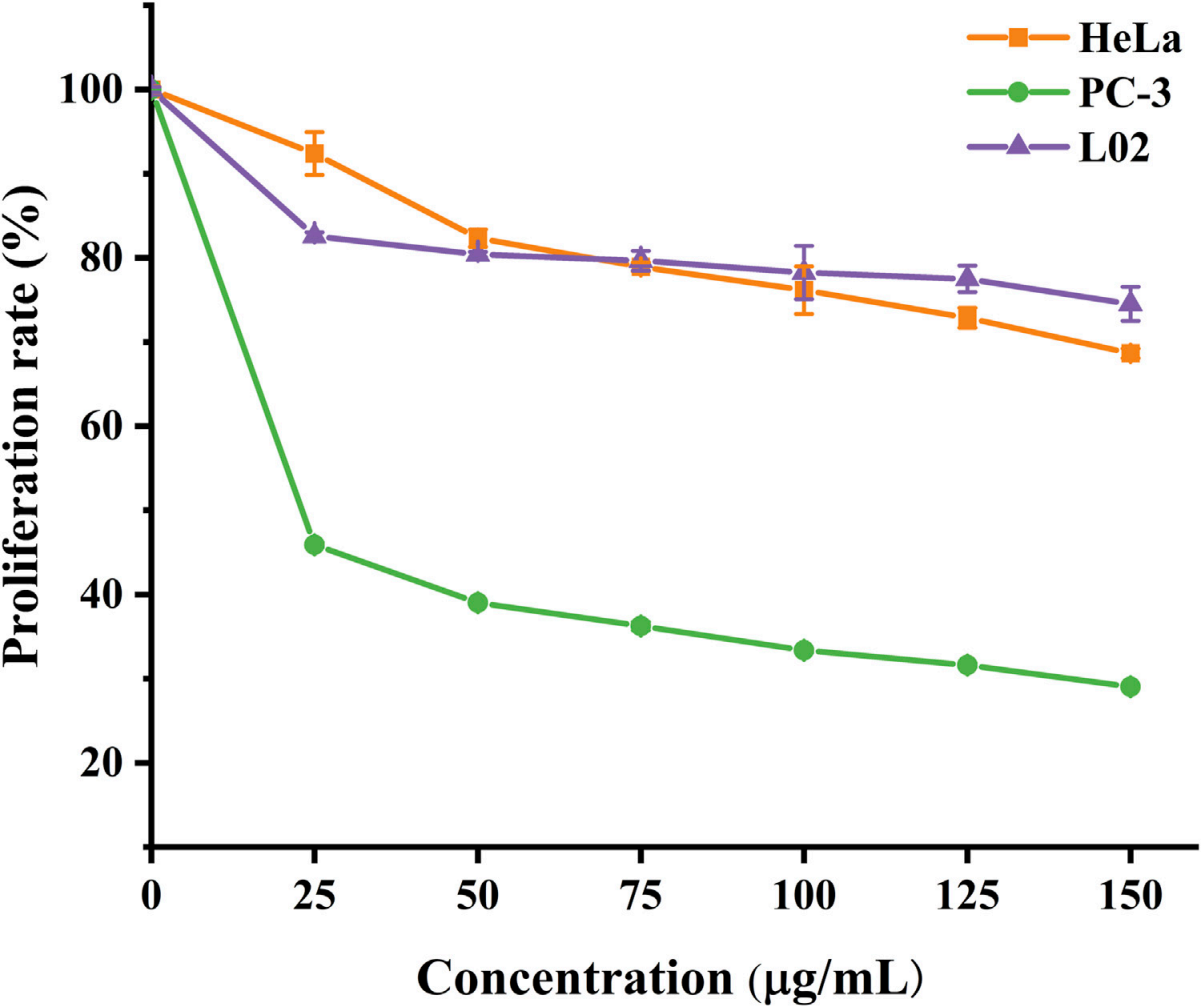
Proliferation rates of HeLa, PC-3 and L02 cells treated with different concentrations of **A-Pind- C**.

Using the CCK8 assay, we evaluated the cytotoxicity of A-PInd-C and selected cancer cells HeLa, PC- 3, and normal cells L02 for the study. According to Figure 12 and Table 3, we observed that the proliferation of all three types of cells was inhibited to a certain extent, with the most significant inhibition rate on PC-3 cells. As the concentration of the compounds increased, the degree of inhibition of A-PInd-C on HeLa and PC-3 cells gradually increased. When the concentration reached 150 ug/mL, the inhibition rates of HeLa and PC-3 cells were 31% and 70%, respectively. However, the degree of inhibition of A-PInd-C on L02 cells hardly changed with the change of concentration, and the inhibition rate remained around 20%. This indicates that A-PInd-C has a relatively small effect on normal cells while having a large inhibitory effect on cancer cells.

**TABLE 3:** Proliferation rate (percentage) of HeLa, PC-3 and L02 cells treated with **A-Pind-C**.

	Blank	25 μg/mL	50 μg/mL	75 μg/mL	100 μg/mL	125 μg/mL	150 μg/mL
HeLa	100±0.4%	92.4±2.5%	82.4±1.0%	78.9±0.5%	76.2±2.9%	72.9±1.2%	68.7±0.6%
PC-3	100±0.1%	45.9±0.3%	39.0±0.2%	36.2±0.5%	33.4±0.3%	31.6±0.3%	29.0±0.2%
L02	100±0.3%	82.6±0.5%	80.4±0.3%	79.7±1.2%	78.3±3.2%	77.5±1.6%	74.5±.0%

## 4 Conclusion

In this study, a polyindole structured polymer was designed and synthesized to load *nido*-carborane and four polymeric nanomaterials were synthesized to encapsulate it. A series of novel fluorescent carborane complexes have been prepared. These complexes not only have good biocompatibility, but also allow the intracellular distribution of drugs to be observed by fluorescence imaging. By UV and fluorescence spectroscopy, it was found that the absorbance and fluorescence intensity of A-PInd-C varied with pH, and had excellent photophysical properties under acidic environment. TEM observed that it showed a spherical stacking in the interior, and AFM imaging showed that the surface of the sample was mostly homogeneous and smooth. The drug can be stably released up to 80% in an acidic environment. Cellular imaging showed that A-PInd-C could enter cancer cells, especially under acidic environment. Cell proliferation toxicity test showed that its inhibitory effect on normal cells basically did not change with the increase of drug concentration, and it had excellent anti-tumor proliferation activity on two cancer cells, especially PC-3 cells, whose inhibition rate reached up to 70%. By improving boron-containing compounds, the system has the potential of BNCT. In addition, the system can improve the function of nanomedicine by encapsulating polymer materials. It provides a theoretical basis for the development and application of carborane fluorescence complexes in the future.

## Data Availability

The original contributions presented in the study are included in the article/Supplementary Material, further inquiries can be directed to the corresponding author.
